# Author Correction: Elective nodal irradiation mitigates local and systemic immunity generated by combination radiation and immunotherapy in head and neck tumors

**DOI:** 10.1038/s41467-024-52861-x

**Published:** 2024-10-10

**Authors:** Laurel B. Darragh, Jacob Gadwa, Tiffany T. Pham, Benjamin Van Court, Brooke Neupert, Nicholas A. Olimpo, Khoa Nguyen, Diemmy Nguyen, Michael W. Knitz, Maureen Hoen, Sophia Corbo, Molishree Joshi, Yonghua Zhuang, Maria Amann, Xiao-Jing Wang, Steven Dow, Ross M. Kedl, Von Samedi, Mary-Keara Boss, Sana D. Karam

**Affiliations:** 1 Department of Radiation Oncology, University of Colorado Denver at Anschutz Medical Campus, Aurora, CO USA; 2 Department of Immunology and Microbiology, University of Colorado Denver at Anschutz Medical Campus, Aurora, CO USA; 3 Department of Otolaryngology Head and Neck Surgery, University of Colorado Denver at Anschutz Medical Campus, Aurora, CO USA; 4 Department of Pathology, University of Colorado Denver at Anschutz Medical Campus, Aurora, CO USA; 5 Department of Pharmacology, University of Colorado Denver at Anschutz Medical campus, Aurora, CO USA; 6 Department of Pediatrics, Cancer Center Biostatistics Core, University of Colorado Anschutz Medical campus, Aurora, CO USA; 7 Roche Innovation Center Zurich, Roche Pharmaceutical Research and Early Development (pRED) Schlieren, Zurich, Switzerland; 8 Department of Pathology and Laboratory Medicine, University of California Davis, School of Medicine, Davis, USA; 9 Veterans Affairs Medical Center, VA Eastern Colorado Health Care System, Aurora, CO 80045 USA; 10 Department of Radiation Oncology, Colorado State University, Fort Collins, Colorado Campus, Aurora, CO USA

**Keywords:** Cancer immunotherapy, Cancer microenvironment, Tumour immunology, Radiotherapy

**Correction to:** *Nature Communications,* 10.1038/s41467-022-34676-w, published online 16 November 2022

The original version of the manuscript contained an error in Supplementary Fig. 8c. In the original figure, “*Tumor only:ENI*” was mistakenly indicated as label for the heatmap in Supplementary Fig. 8c. Instead, this should have been labeled as “*ENI:Tumor Only*”. Supplementary Fig. 8 has been corrected in the updated version of the Supplementary Information file now available. The related Source Data have been also revised accordingly in the newly uploaded Source Data file.

In addition, in the article file, the sentence describing the figure above was also inaccurate and has now been corrected.

The original sentence in the Results section *Tumor only SBRT increases immune responses in canines and humans with HNSCC*, read as “*All these genes associated with an increase in antigen presentation (MHC I and/or MHC II antigen presentation) were decreased in dogs that received ENI. Activation genes associated with effector T cells (GZMB and IFNG) and CXCL10, a cytokine associated with increased T cell homing, were also decreased in ENI treated dogs (Supplementary Fig. 8C)”*.

The sentence has now been rephrased as “*All these genes associated with an increase in antigen presentation (MHC I and/or MHC II antigen presentation) were* increased *in dogs that received ENI. Activation genes associated with effector T cells (GZMB and IFNG) and CXCL10, a cytokine associated with increased T cell homing, were also* increased *in ENI-treated dogs (Supplementary Fig. 8C). On the other hand, genes associated with immunosuppression (FOXP3, IL10RA, and IL17RB) were* decreased *in dogs treated with ENI (Supplementary Fig. 8C). However, none of the genes associated with antigen presentation, T cell activation, or immunosuppression were significantly different between dogs that received ENI and those that did not (Supplementary Fig. 8). Although we did not find any significant differences in gene expression in the LN, irradiation of the lymph nodes translated into a decrease in T cells in the TME three days post-RT (Fig. 6D)*.”

The corrections above have now been made in the revised PDF and HTML versions of the article file.

