# Supplementary material for: Elective nodal irradiation mitigates local and systemic immunity generated by combination radiation and immunotherapy in head and neck tumors
Source: Nat Commun. 2022 Nov 16;13:7015. doi: 10.1038/s41467-022-34676-w (PMC9668826; doi:10.1038/s41467-022-34676-w)
Supplement: Supplementary file 1 — Supplementary Information [file 41467_2022_34676_MOESM1_ESM.pdf]

Supplemental Information

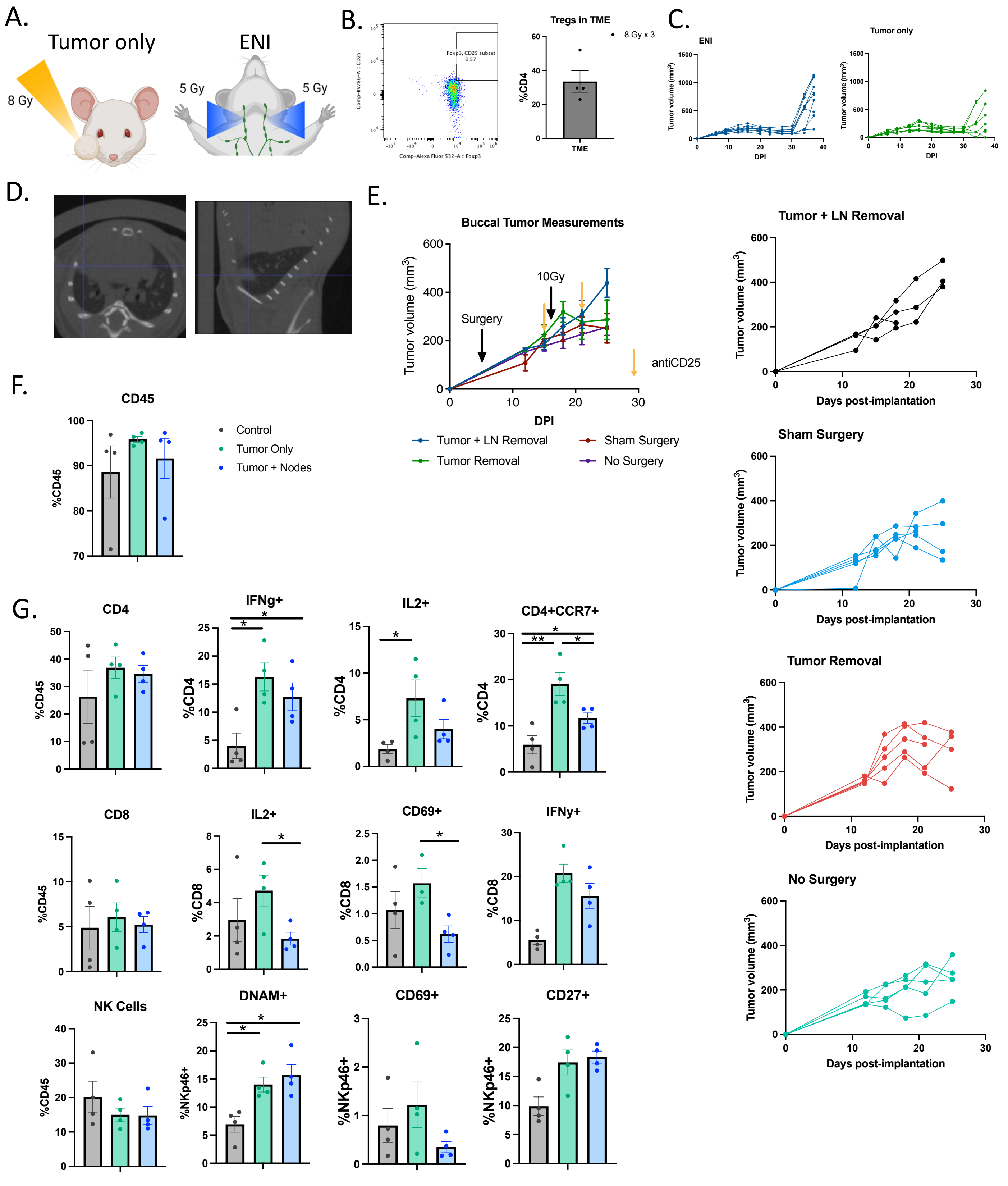

Supplemental Figure 1: ENI ablates the immune response to combined radiation and immunotherapy in HNSCC models. A) Schematic of how SBRT was delivered to the tumor or the bilateral draining lymph nodes

(DLNs). Tumor only mice received 8 Gy x 3 to the buccal tumor (yellow), while mice that were treated with ENI received both buccal (8 Gy x 3, yellow) irradiation and 5 Gy x 3 bilateral nodal irradiation (blue). Created with BioRender.com. B) Representative flow plot of Treg depletion in mice (left) and percentage of Tregs in the TME of mice with LY2 tumors after treatment with 8 Gy x3 (n=4) (right). C) Individual tumor growth curves for the mice with P029 tumors (n=10 for each group). D) Representative images of a lung metastasis in a mouse treated with ENI with P029 tumors at DPI 41. E) Buccal tumor growth curves for mice with LY2 tumors that received neck dissections prior to combined radiation and immunotherapy treatment (Tumor + DLN removal, n=4; Tumor removal, n=5; Sham surgery, n=5, no surgery, n=5). F) Quantification of CD45+ lymphocytes in the blood of mice from the experiment depicted in Figure 1B (n=4 per group). G) Quantification of CD8 T cells, CD4 T cells, and NK cells as percentages of CD45+ cells in the circulation (n=4 per group). For difference between cell percentages in **f.** multiple student's t-tests were used. Significance was determined if the p-value was < 0.05* and <0.01**. All data are reported with mean ± SEM. Source data are provided as a Source Data file. p-values are indicated for figure **g.** $CD4^+IFNg^+$ tumor only *0.01, $CD4^+IFNg^+$ ENI *0.0383, $CD4^+IL\text{-}2^+$ *0.0356, $CD4^+CCR7^+$ **0.0064, $CD4^+CCR7^+$ control vs tumor + nodes *0.0468, $CD4^+CCR7^+$ tumor only vs tumor + nodes *0.0366, $CD8^+IL\text{-}2^+$ *0.0286, CD69 *0.0222, DNAM control vs tumor only *0.0107, DNAM control vs tumor + nodes *0.0105.

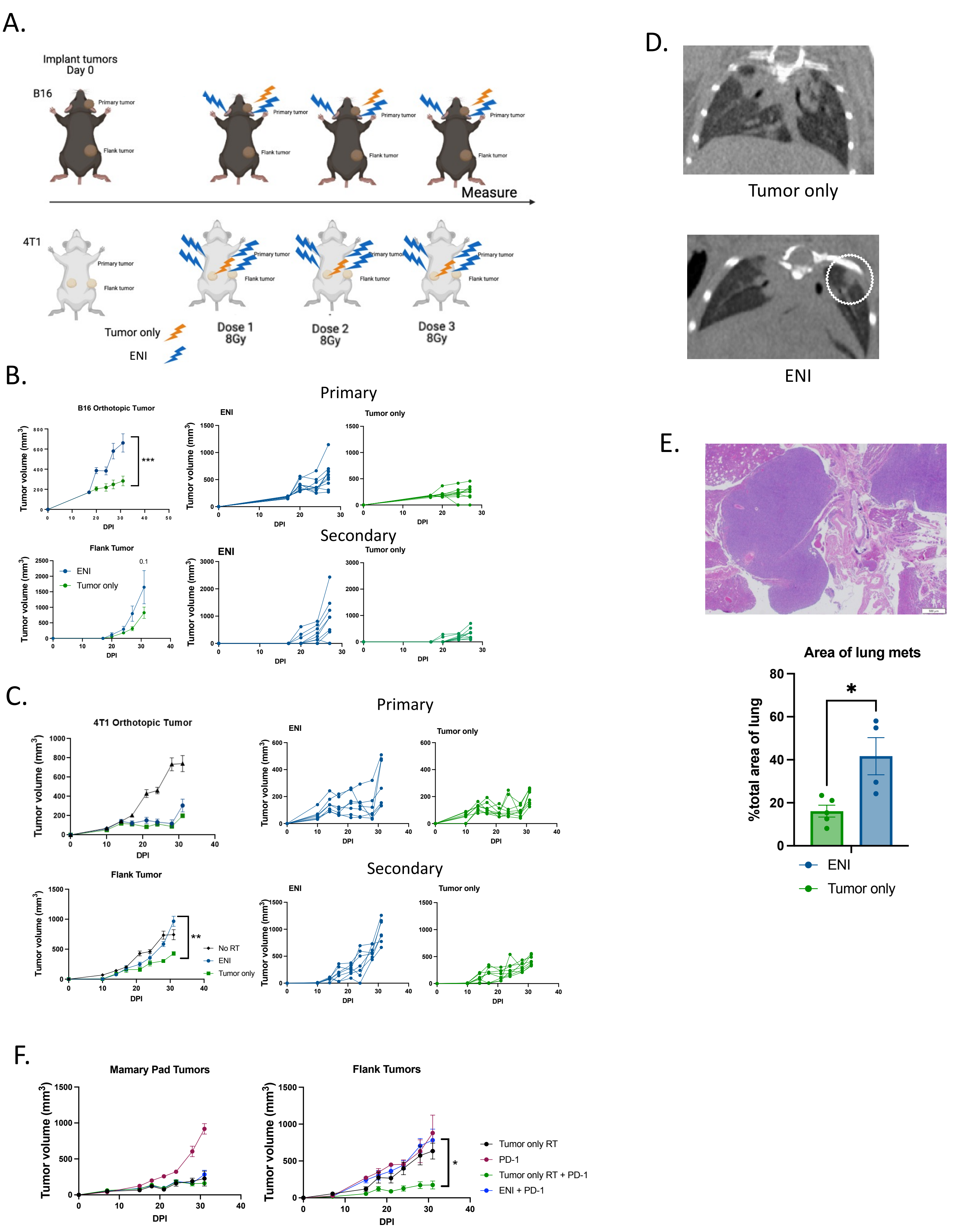
A.
Implant tumors
Day 0
B16
Primary tumor
Flank tumor
Measure
4T1
Tumor only
ENI
Dose 1
8Gy
Dose 2
8Gy
Dose 3
8Gy
B.
B16 Orthotopic Tumor
Primary
ENI
Tumor only
Tumor volume (mm³)
DPI
***
Secondary
Flank Tumor
ENI
Tumor only
0.1
C.
4T1 Orthotopic Tumor
Primary
Secondary
Flank Tumor
No RT
ENI
Tumor only
**
D.
Tumor only
ENI
E.
Area of lung mets
%total area of lung
*
ENI
Tumor only
F.
Mamary Pad Tumors
Flank Tumors
Tumor volume (mm³)
DPI
*
Tumor only RT
PD-1
Tumor only RT + PD-1
ENI + PD-1

Supplemental Figure 2: ENI ablates the immune response to combined radiation and immunotherapy in B16 and 4T1 models. A) Experimental schematic for the B16-OVA and 4T1 ENI experiments. Created with BioRender.com. B) Primary and flank tumor growth curves for mice implanted B16-OVA (ENI, n=10; tumor only, n=9). C) Primary and flank tumors growth curves for mice implanted with the 4T1 cell line (ENI, n=7; tumor only, n=8; No RT, n=10). D) Representative lung microCT images of mice from the 4T1 model. A metastasis in an ENI treated mouse is highlighted (white circle). Quantification of H&E images of lung metastases at time of death (ENI, n=4; tumor only, n=5). E) H&E staining of a representative lung of a mouse treated with ENI in the 4T1 model, 4 ENI and 5 tumor only lungs were stained. F) Primary and flank tumors growth curves for mice implanted with the 4T1 cell line (aPD-1 only, n=4; tumor only alone, n=5; ENI, n=7; tumor only, n=7). For tumor growth at different time points, with two or more groups differences were determined by a One-Way ANOVA test with Tukey's post hoc and a Two-Way ANOVA for comparing two groups. Significance was determined if the p-value was < 0.05* and <0.01**. All data are reported with mean ± SEM. Source data are provided as a Source Data file. p-values are indicated for figures **b.** ***0.0006, **c.** **0.0026, **f.** *0.0468, and **e.** *0.0170.

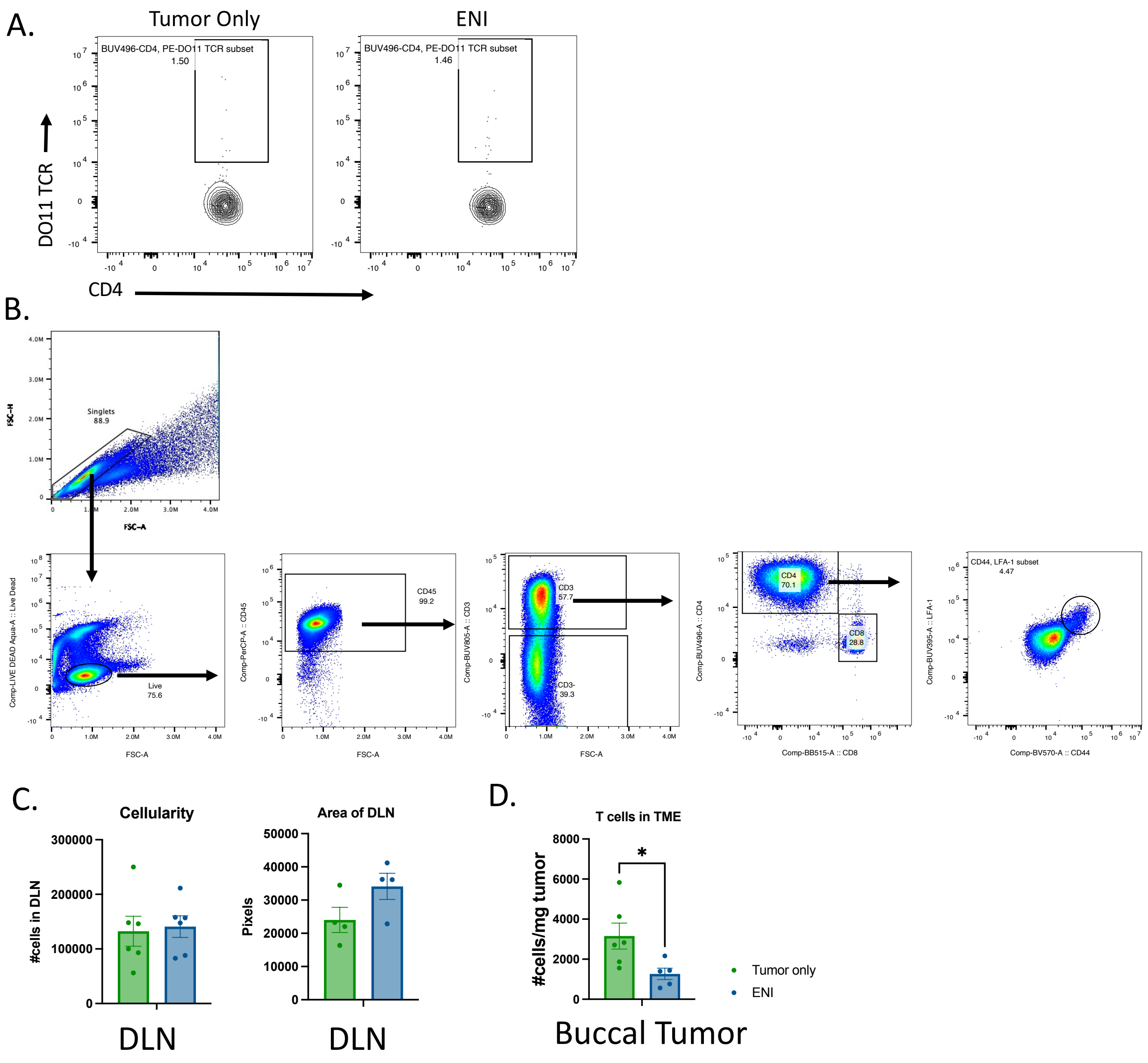

Supplemental Figure 3: ENI decreases antigen-experienced T cell expansion in the DLNs and infiltration into the TME. A) Representative flow gating plots of DO11+ CD4+ T cells in both ENI and tumor only treated mice. B) Gating strategy for LFA-1+CD44+ CD4 T cells which was used for all compartments analyzed. C) Quantification of the number of cells within the DLN (ENI, n=6; tumor only, n=6) and total area of DLNs (ENI, n=4; tumor only, n=4) of mice treated with ENI and mice treated with tumor only SBRT at the time of flow cytometry, 3 days post the last dose of SBRT. Cells were quantified using InForm software and Akoya Phenoptoreports and DLN total area was quantified using Fiji (ImageJ). D) Quantification of T cells (CD3+) in the buccal tumor TME per mg of tumor (ENI, n=5; tumor only, n=6). A two-tailed student's t-test was used to determine significance. Significance was determined if the p-value was < 0.05*. The error bars represent the

standard error of the mean (± SEM)**.** Source data are provided as a Source Data file. p-values are indicated for figure **d.** *0.0349.

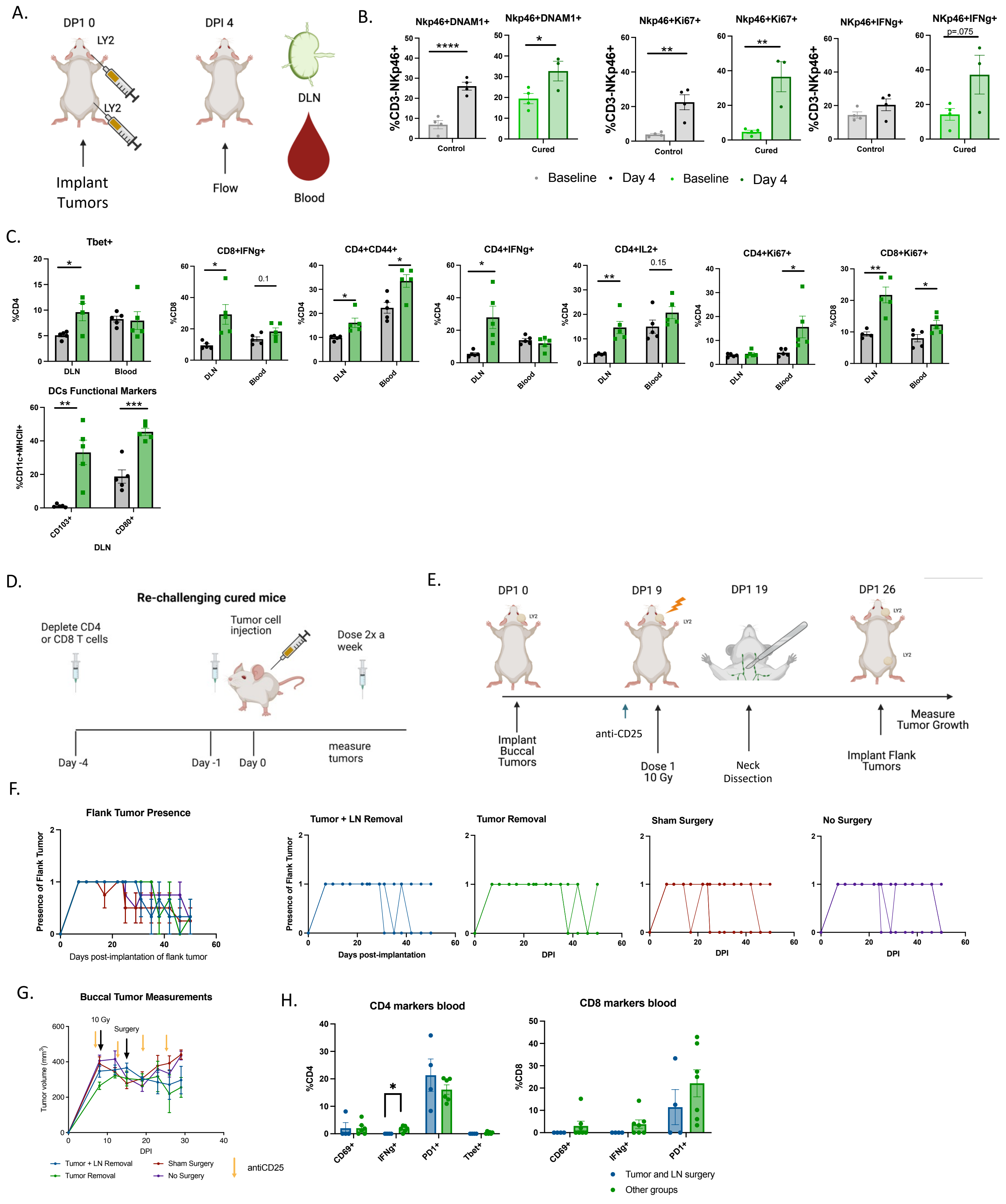

Supplemental Figure 4: Systemic, long-term, DLN-independent memory is formed with tumor only SBRT. A) A schematic of the experimental design of re-challenging cured mice and harvesting DLNs and

blood 4 days post-implantation (<6 months post tumor eradication, mice were <1 year of age). Created with BioRender.com. B) Quantification of NK cells in the DLN and in the blood of mice rechallenged with LY2 cells in the buccal or naïve mice (Cured, n=4; Naïve, n=4). C) Mice cured of LY2 tumors with tumor only SBRT and anti-CD25 (>1 year previously, mice were greater than 1.5 years old) and naïve mice (n=5 in both groups) were rechallenged with LY2 tumor cells as in Supplemental Figure 4A. Immune cells in the DLN and blood were quantified. D) Schematic of how mice were rechallenged with LY2 with CD4 or CD8 T cell depletion compared to naïve mice implanted with LY2 cells. Mice were depleted of CD4 or CD8 T cells before tumor implantation (DPI -4 and -1). Created with BioRender.com. E) Schematic of the delayed surgical neck dissection experiment. LY2 tumors were implanted (DPI 0). Once tumors reached ~150mm^3 the mice were given anti-CD25 and then dosed with 10 Gy of SBRT the next day. Ten days later, the mice underwent tumor and LN dissection, tumor dissection, a sham surgery, or had no surgery. Seven days after surgery, flank tumors were implanted and monitored for growth. Created with BioRender.com. F) Presence of flank tumors from the mice undergoing a delayed neck dissection (Tumor + LN removal, n=6; Tumor removal, n=6; Sham surgery, n=6; and no surgery, n=4). G) Tumor measurements of mice that underwent delayed neck dissection that were used for flow cytometry (Tumor + LN removal, n=5; Tumor removal, n=4; Sham surgery, n=3; and no surgery, n=5). H) Quantification of flow cytometry data of T cells in the blood of mice that underwent a delayed neck dissection (LN resection, n=4; all other groups, n=6-7). A two-tailed student's t-test was used to determine the significance of differences between the two groups. Significance was determined if the p-value was < 0.05*, <0.01**, <0.001***, and <0.0001****. The error bars represent the standard error of the mean (± SEM). Source data are provided as a Source Data file. p-values are indicated for figures **b.** DNAM ****<0.0001, DNAM *0.0438, control Ki67 **0.0054, cured Ki67 **0.0075, **c.** Tbet *0.0205, CD8$^+$IFNg$^+$ *0.0148, CD44 DLN *0.0144, CD44 blood *0.0124, CD4$^+$IFNg$^+$ *0.0118, IL-2 **0.0064, CD4$^+$Ki67$^+$ *0.048, CD8$^+$Ki67$^+$ DLN **0.0036, CD8$^+$Ki67$^+$ blood *0.0382, CD103 **0.0024, CD80 ***0.0004, and **h.** *0.0151.

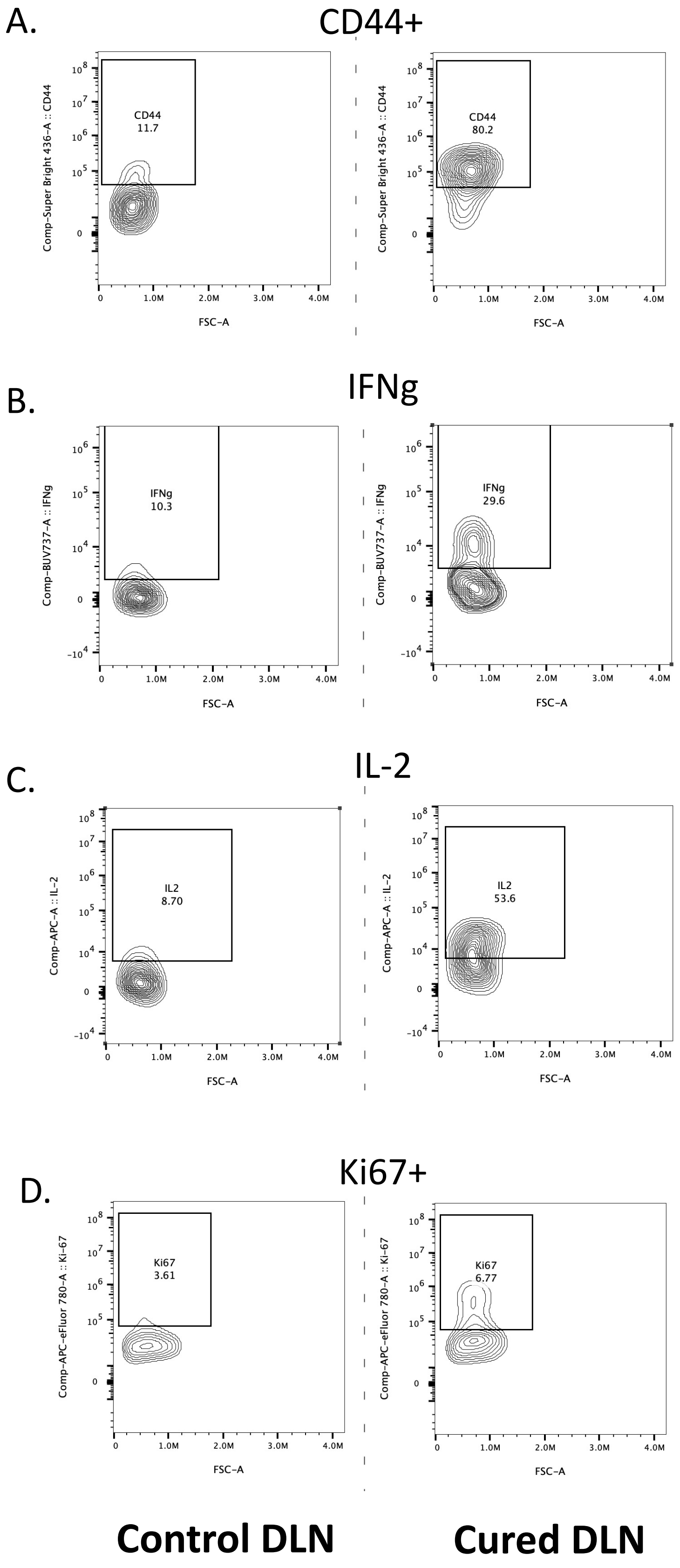

Supplemental Figure 5: Gating Strategies for activation Markers on T cells. A-D) Representative flow cytometry gating in FlowJo for activation markers shown in Figure 3.

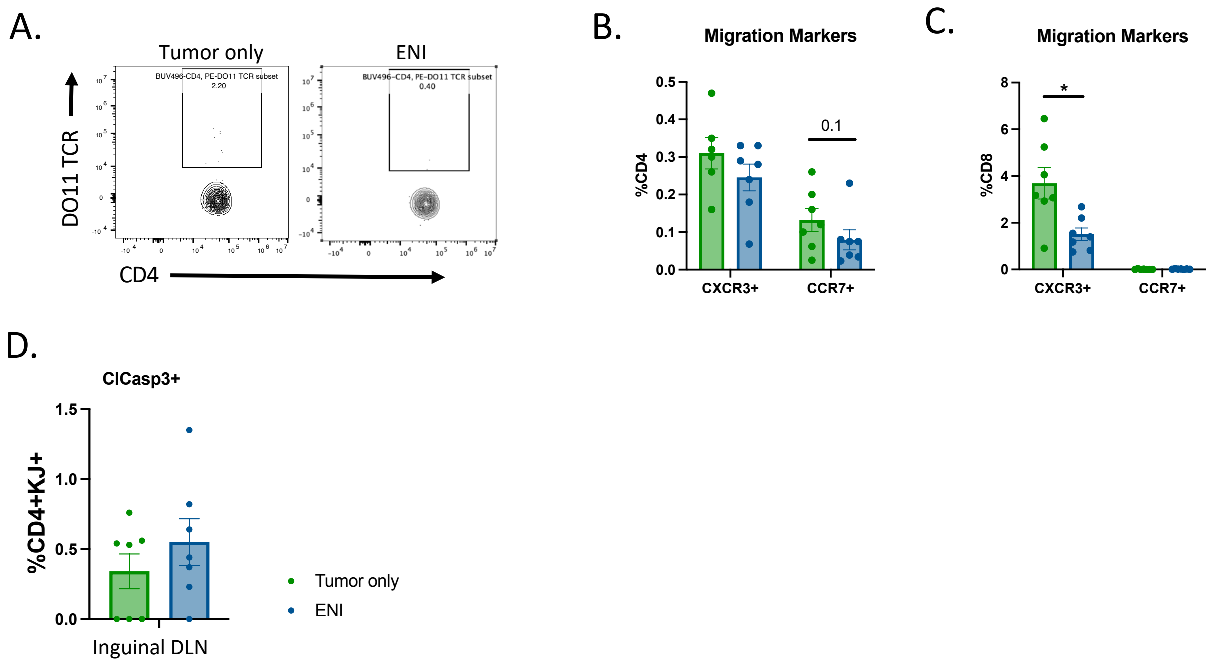

Supplemental Figure 6: Migration markers expressed on T cells and apoptosis marker expressed on T cells in the DLNs. A) Gating strategy for KJ+ CD4 T cells in the blood of mice treated with ENI or tumor only SBRT depicted in Figure 4A. B-C) CXCR3 and CCR7 expression on CD4 (B) and CD8 (C) T cells in the draining lymph node of mice treated with tumor only SBRT or ENI (ENI, n=7; tumor only, n=7). D) Expression of cleaved caspase 3 in the inguinal node of mice treated with or without ENI (ENI, n=7; tumor only, n=7). A two-tailed student's t-test was used to determine the significance of differences between the two groups. Significance was determined if the p-value was < 0.05*. The error bars represent the standard error of the mean (± SEM). Source data are provided as a Source Data file. p-values are indicated for figure **c.** *0.0111.

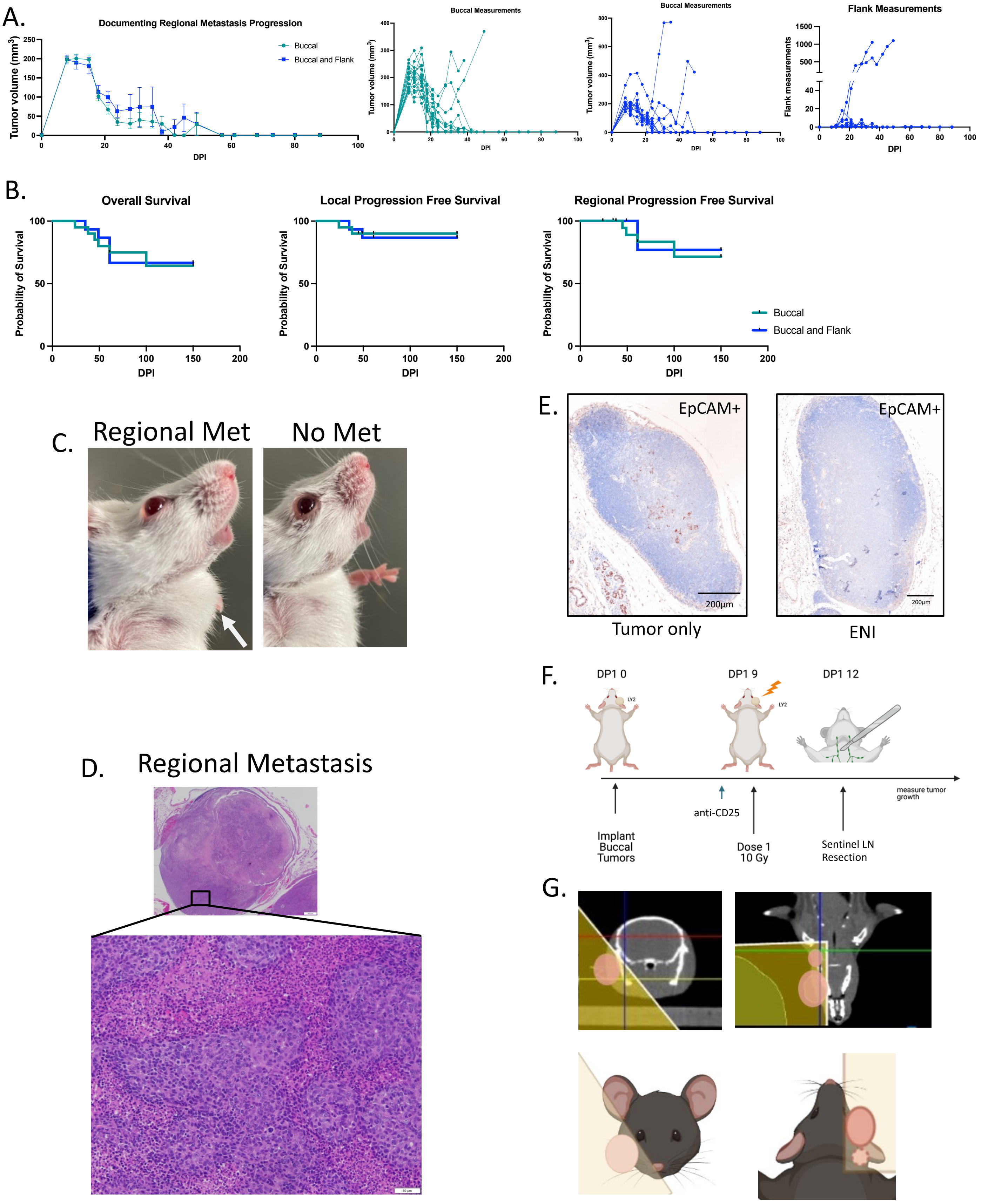

Supplementary Figure 7: Sentinel node resection, or irradiation, reduces regional recurrence. A) Buccal tumor growth curves for mice implanted with either a buccal tumor or with a buccal and flank tumor and treated

with 10 Gy x1 and anti-CD25. Individual growth curves of buccal tumors and the flank tumors are included to the right (buccal only, n=15; buccal and flank, n=10). B) Overall survival, local progression free survival and regional progression free survival curves comparing mice implanted with either a buccal tumor or a buccal tumor and a flank tumor (buccal only, n=15; buccal and flank, n=10). C) Representative images of a mouse with (left) and without (right) a regional metastasis. D) Representative image of an H&E of a regional metastasis and a 20x image of a regional metastasis. 5 nodal metastases were stained. E) Representative images of sentinel lymph nodes stained for EpCAM of mice treated with or without ENI from the experiment depicted in Figure 2B. Sentinel lymph nodes were harvested at the same time as cells were for flow cytometry. 4 ENI and 5 tumor only nodes were stained. F) Experimental schematic for the sentinel lymph node resection experiment. Buccal tumors were implanted on DPI 0. Mice were treated with 10 Gy x 1 and anti-CD25 starting the day before SBRT and weekly afterwards. Mice underwent a sham surgery or sentinel lymph node resection on DPI 12 (n=15 for both groups). Created with BioRender.com. G) Experimental schematic for sentinel lymph node irradiation. Created with BioRender.com and SmART + ATP Plan, Precision X-Ray, Inc. (Madison). The error bars represent the standard error of the mean (± SEM). Source data are provided as a Source Data file.

A.

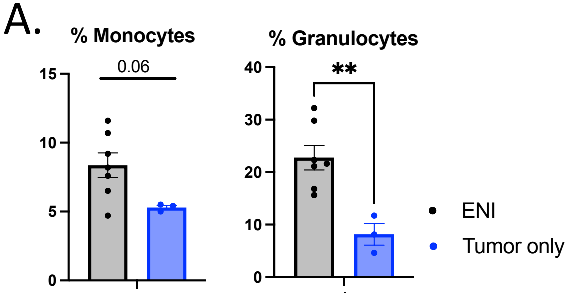
% Monocytes
0.06
15
10
5
0
% Granulocytes
**
40
30
20
10
0
ENI
Tumor only

B.

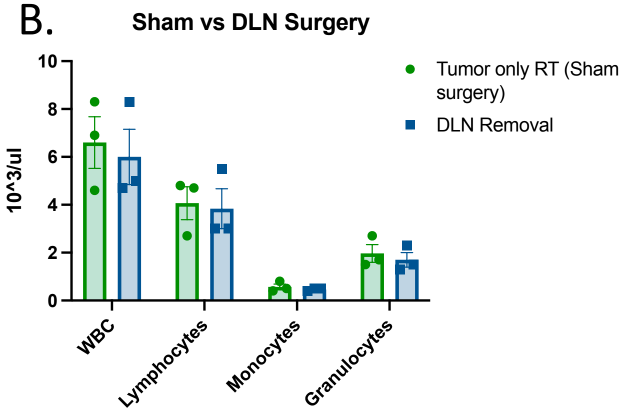
Sham vs DLN Surgery
10^3/ul
10
8
6
4
2
0
WBC
Lymphocytes
Monocytes
Granulocytes
Tumor only RT (Sham surgery)
DLN Removal

C.

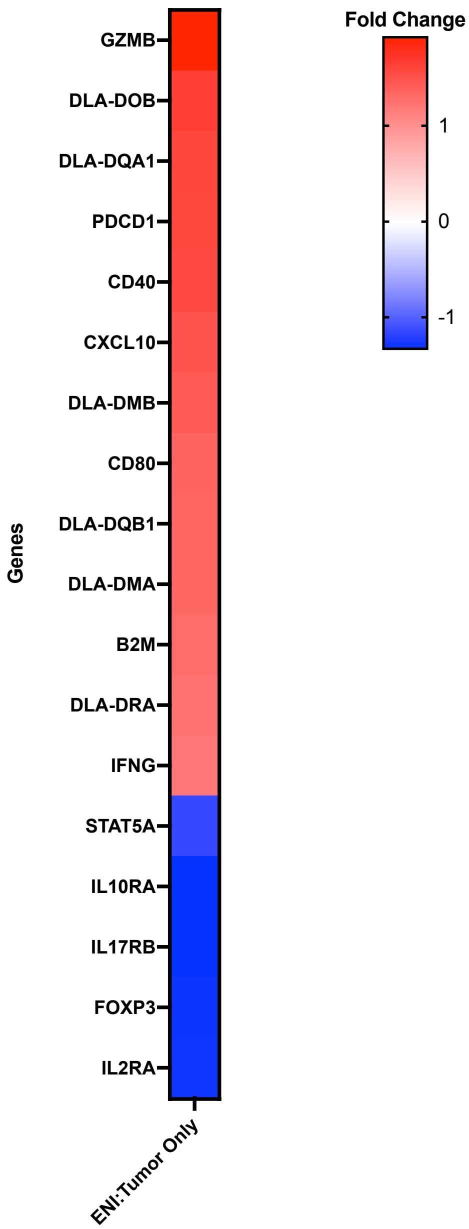
Genes
GZMB
DLA-DOB
DLA-DQA1
PDCD1
CD40
CXCL10
DLA-DMB
CD80
DLA-DQB1
DLA-DMA
B2M
DLA-DRA
IFNG
STAT5A
IL10RA
IL17RB
FOXP3
IL2RA
ENI:Tumor Only
Fold Change
1
0
-1

Supplemental Figure 8: Tumor only SBRT increases immune responses in canines and humans with HNSCC. A) Blood was collected from B16-OVA implanted mice treated with ENI or tumor only SBRT. Percentages of monocytes and granulocytes were quantified (ENI, n=7; tumor only, n=3). B) Blood was collected from LY2 implanted mice treated with tumor only SBRT and a sham surgery or sentinel lymph node removal and white blood cells (WBCs), lymphocytes, monocytes and granulocytes were quantified (Sham surgery, n=3; DLN removal, n=3). C) Heatmap of the fold change in gene expression of genes related to antigen presentation, T cell activation, and immunosuppression in LNs of dogs treated with or without ENI (n=3 for both groups). A two-tailed student's t-test was used to determine the significance of group differences. Significance was determined if the p-value was < 0.05* and <0.01**. The error bars represent the standard error of the mean (± SEM). Source data are provided as a Source Data file. p-values are indicated for figure **a.** **0.0056.
